# Cellular dissection of malaria parasite invasion of human erythrocytes using viable *Plasmodium knowlesi* merozoites

**DOI:** 10.1038/s41598-018-28457-z

**Published:** 2018-07-05

**Authors:** Oliver Lyth, Gema Vizcay-Barrena, Katherine E. Wright, Silvia Haase, Franziska Mohring, Adrian Najer, Isabelle G. Henshall, George W. Ashdown, Lawrence H. Bannister, Damien R. Drew, James G. Beeson, Roland A. Fleck, Robert W. Moon, Danny W. Wilson, Jake Baum

**Affiliations:** 1 Department of Life Sciences, Imperial College London, Sir Alexander Fleming Building, Exhibition Road, South Kensington, London, UK; 2 Centre for Ultrastructural Imaging, Guy’s Campus, King’s College London, London, UK; 3 Department of Immunology and Infection, London School of Hygiene and Tropical Medicine, London, UK; 4 Research Centre for Infectious Diseases, School of Biological Sciences, The University of Adelaide, Adelaide, Australia; 5 Burnet Institute, 85 Commercial Road, Melbourne, Victoria, Australia; 6 Central Clinical School, Monash University, Victoria, Australia

## Abstract

*Plasmodium knowlesi*, a zoonotic parasite causing severe-to-lethal malaria disease in humans, has only recently been adapted to continuous culture with human red blood cells (RBCs). In comparison with the most virulent human malaria, *Plasmodium falciparum*, there are, however, few cellular tools available to study its biology, in particular direct investigation of RBC invasion by blood-stage *P*. *knowlesi* merozoites. This leaves our current understanding of biological differences across pathogenic *Plasmodium* spp. incomplete. Here, we report a robust method for isolating viable and invasive *P*. *knowlesi* merozoites to high purity and yield. Using this approach, we present detailed comparative dissection of merozoite invasion (using a variety of microscopy platforms) and direct assessment of kinetic differences between *knowlesi* and *falciparum* merozoites. We go on to assess the inhibitory potential of molecules targeting discrete steps of invasion in either species via a quantitative invasion inhibition assay, identifying a class of polysulfonate polymer able to efficiently inhibit invasion in both, providing a foundation for pan-*Plasmodium* merozoite inhibitor development. Given the close evolutionary relationship between *P*. *knowlesi* and *P*. *vivax*, the second leading cause of malaria-related morbidity, this study paves the way for inter-specific dissection of invasion by all three major pathogenic malaria species.

## Introduction

Pathology associated with malaria disease arises from *Plasmodium* parasite infection and replication within red blood cells (RBCs). This stage begins when the micron-sized *Plasmodium* merozoite, responsible for blood stages of infection, drives its own internalization into the RBC (reviewed in^1^). The invaded parasite then concomitantly establishes itself for growth and development within a membrane-lined parasitophorous vacuole, replicating, egressing and initiating further rounds of infection^2^.

Broadly conserved mechanisms are thought to underlie this process of merozoite entry into the RBC across *Plasmodium* spp. that infect humans^1^. There are, however, important known differences in parasite ligand-host cell receptor interactions used^3^. Precise resolution of these differences has been hampered by failed attempts at adapting the major human malaria parasites to culture, with the notable exception of *P*. *falciparum*, successfully adapted to culture in the 1970s^4^. Attempts to culture-adapt *P*. *vivax*, the other major cause of global malaria morbidity, have not met with similar success largely because of its preference for the minority population of circulating immature reticulocytes^5^. As such, dissection of the precise cellular and molecular differences between species has been only slowly forthcoming^6^.

The zoonotic malaria parasite *P*. *knowlesi* has recently been culture-adapted to human RBCs^7–9^, providing a means to explore the biology of this emerging human pathogen^10^ and compare invasion biology with established *P*. *falciparum* parasite lines. Furthermore, its close phylogenetic relationship to *P*. *vivax* makes *P*. *knowlesi* an ideal platform for comparative invasion biology across *Plasmodium spp*., with study of its invasion into human RBCs becoming an increasingly important area of parasite blood-stage research^11^. One remaining road-block to its use, however, is the apparent fragility of the merozoite lifecycle stage and difficulties associated with purifying enough viable cells to undertake downstream experiments. This bottleneck was overcome for *P*. *falciparum* with the development of a method for isolation of viable merozoites^12^, enabling their application to studying dynamic events during RBC invasion^13–16^, therapeutics that inhibit entry^17–19^ and the immune response targeting merozoite antigens^20–22^. The same methodological process has not been available for other *Plasmodium* species, with the exception of murine malaria species *P*. *berghei*^17,23,24^ and *P*. *yoelii*^25^, which can only be isolated after harvesting high parasitemia, *in vivo-*derived infected blood.

Previous reports have described isolation of *P*. *knowlesi* merozoites from parasite-infected macaque RBCs by filtration through a polycarbonate sieve (e.g. refs^26–29^) or following mechanical release of merozoites from mature schizonts through a syringe needle^30,31^. These methods require bespoke apparatus (no longer available) or involve harvesting over a prolonged period (2–3 hrs), affecting cell viability. In addition, their reliance on simian-infected RBCs, has limited their broad reproducibility. Here, we address this gap in our technological armamentarium by developing a robust methodological workflow to routinely isolate large numbers of high purity, viable (i.e. invasive) human tissue culture-adapted *P*. *knowlesi* merozoites. We use these merozoites to dissect the cell biology of invasion in *P*. *knowlesi,* characterise inhibitory antibodies and compounds that block invasion, while identifying novel therapeutics that target parasite entry. Run in parallel with *P*. *falciparum* isolated merozoites, this approach lays the foundations for detailed comparative RBC invasion biology across *Plasmodium* species.

## Results

### Isolation of viable and invasive *P*. *knowlesi* merozoites

We sought to establish a robust, high yielding and reproducible process for isolation of invasive blood-stage merozoites from human RBC culture-adapted *P*. *knowlesi*. *P*. *knowlesi* ring stage parasites were allowed to develop through to schizogony using tightly synchronous cultures from either a Nycodenz gradient or addition of heparin (A1.H-1 and YH1 strains, respectively). At schizogony (24 hrs [A1-H.1] or 28 hrs [YH1] hrs post invasion) parasites were purified from uninfected RBCs by magnet separation and incubated with the cysteine protease inhibitor E64 for no more than 4 hrs, inhibiting rupture of the RBC membrane and parasite egress^12,32^, until fully formed segmented-schizonts were found in the majority of infected RBCs (Fig. S1).

**Figure 1:**
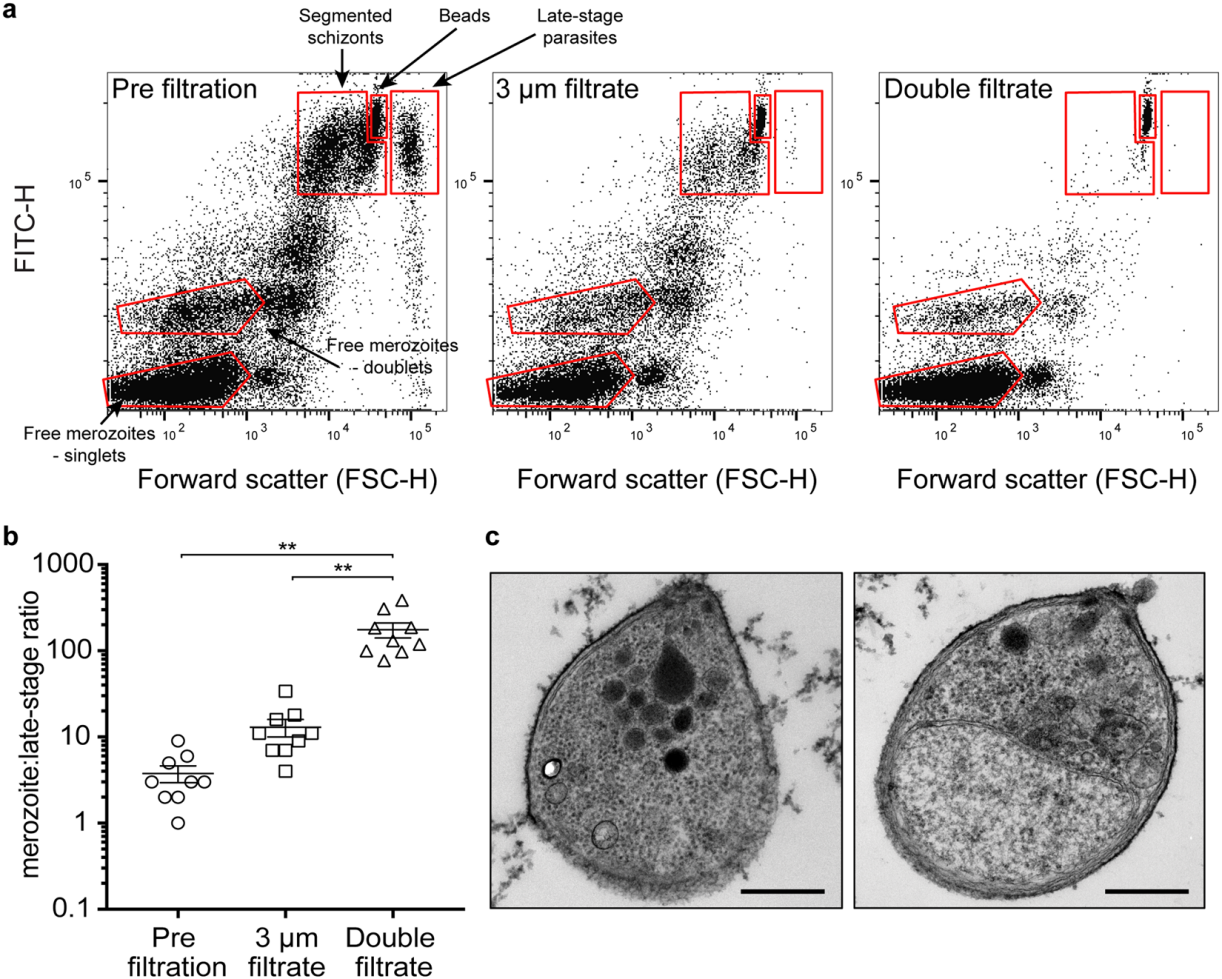
Double filtration of A1-H.1 *P*. *knowlesi* E64-arrested schizonts isolates merozoites of high purity. (**a**) Representative flow cytometry plots showing different cell populations, pre and post filtration. Plots do not show uninfected RBCs (y-axis has been cropped to give better resolution of free merozoite populations). Figure S3 shows uncropped RBC population. Free merozoite population was tightly-gated after identification of haemozoin-derived events (Fig. S1). (**b**) Double-filtration increases the merozoite:late-stage ratio significantly, giving a free merozoite preparation of high purity. Graph shows mean from n = 9 experiments. Error bars represent SEM. P-values comparing different merozoite:late-stage ratios represent one-way multiple comparisons ANOVA test (**P < 0.01). (**c**) TEM image of free merozoites. Scale bars = 500 nm.

*P*. *knowlesi* merozoites are larger (~2–3 μm) than their *P*. *falciparum* counterparts (~1–1.5 μm^33^) precluding use of 1.2 µm (as used for *P*. *falciparum* merozoite purification^12^) or 1.6 µm filters. Use of 5 μm filters yielded poor purity allowing a high proportion of late-stage parasites and uninfected RBCs to pass through largely undisturbed. Syringe mediated filtration with a 3 μm filter by itself also showed high levels of late stage parasite contamination (Fig. 1a). Trialling double filtration of E64-arrested schizont cultures, we identified that passage using a combined 3 μm and subsequent 2 μm filter set successfully provided a merozoite preparation of high purity with significantly reduced numbers of contaminating infected and uninfected RBCs (Figs 1a,b and S1). Double filtration led to a >10-fold increase in the ratio of merozoites to late-stage schizonts compared to use of a single 3 μm filter (Fig. 1b). The final filtrate contained merozoites of high purity along with haemozoin crystals. Haemozoin and any remaining contaminating schizonts could subsequently be removed by a further magnet purification step (Fig. S1), yielding pure merozoite preparations if needed. We found that a 15–20 μl schizont pellet (from 50 ml parasite culture, 2% hematocrit, 2% parasitemia) resuspended in 1.5 ml incomplete media and double filtered gave on average a yield of 1.10 × 10^8^ ± 0.12 × 10^8^ merozoites/ml (mean ± SEM, 12 experiments). We demonstrated that the purified free merozoites were suitable for transmission electron microscopy (TEM) and that broad cell architecture remained intact (Fig. 1c).

We next sought to confirm whether *P*. *knowlesi* merozoites retained invasive capacity post-filtration. Upon incubation of double filtrate of either purified A1.H-1 or YH1 merozoites with fresh human RBCs in suspension cultures, a proportion of merozoites showed rapid invasion with ring-stage parasites visualised within <1 hour (Fig. S2). *P*. *knowlesi* parasites that invaded after purification underwent normal development in a highly synchronous culture as exemplified by visualisation of 12-hour trophozoite stage parasites (Fig. S2).

### Analysis of invasion kinetics of *P*. *knowlesi* merozoites following filtration

**Figure 2:**
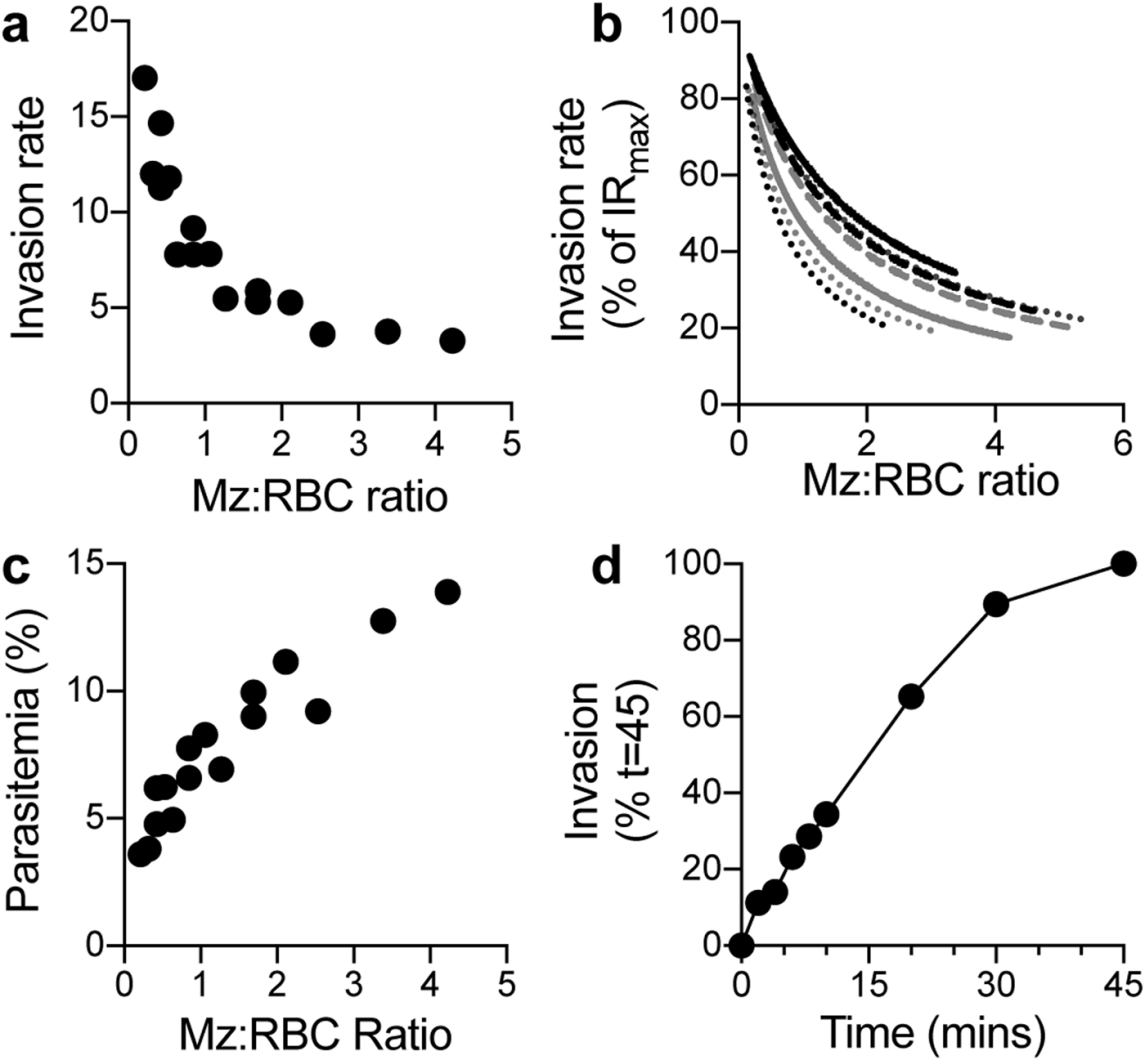
Invasion kinetics of *P*. *knowlesi* filter isolated merozoites. (**a**) Proportion of merozoites invading RBCs (invasion rate) is influenced by ratio of merozoites:RBCs. Data are representative of n = 7 assays in duplicate. (**b**) Merozoite:RBC ratio giving 50% maximum invasion rate (IR_50_). For each experiment the highest invasion rate was normalized to 100% (IR_max_) and invasion rates at all other ratios expressed as a proportion of IR_max_ (n = 7 experiments shown). (**c**) Increasing the merozoite:RBC ratio leads to an increase in parasitemia (n = 7 assays in duplicate shown). (**d**) The proportion of merozoites that have invaded with increasing time shown as % of invasion after 45 mins (mean of n = 3 experiments in duplicate). Error bars of SEM are not visible as they are shorter than height of symbols.

We next used purified A1.H-1 *P*. *knowlesi* merozoite filtrate and diluted RBCs by flow cytometry (Figs 1a and S3) to assess the kinetics of their invasion capacity. Both merozoite and RBC concentrations were varied independently to determine effect on efficiency of invasion. Maximum invasion rates (proportion of the total number of parasites invaded), and therefore maximum viability of merozoites, was achieved at low merozoite:RBC ratios where RBCs are in excess (Fig. 2a). The IR_50_ (merozoite:RBC ratio that gives half maximum invasion rate IR_max_) was calculated to be 1.19 ± 0.17 (mean ± SEM, 7 experiments) (Fig. 2b). Maximum invasion efficiencies achieved ranged from 5–6% through to 17%, lower than the 25–30% multiplication rate typical for *P*. *knowlesi* during *in vitro* culture with human RBCs. Of note, the IR_50_ and multiplication rate found were similar to those previously described for *P*. *falciparum* purified merozoites^12^. However, under these conditions, parasitemia achieved was low and therefore was not appropriate for inhibitor studies by flow cytometry or fixing invasion events for imaging. To achieve the highest parasitemia, an excess of merozoites was added to RBCs (Fig. 2c) (yielding parasitemia of 20.7%, 13.9% and 12.5% for the three best experiments). Balancing between invasion rate and parasitemia, the majority of the work completed here was done at 0.5–1% hematocrit to achieve a higher parasitemia required for downstream analysis.

We next explored invasion inhibition at different time-points to determine the proportion of successful invasion events over time *in vitro*. For *P*. *falciparum*, 80% of maximal invasion occurs in the first 10 mins^12^. Remarkably, for *P*. *knowlesi* merozoites, only 35% of total invasion events occurred during this window of time (Fig. 2d) with invasion continuing steadily up to 30 mins (90% of total invasion events) after addition of merozoites to RBCs. Of note, agitation (500–750 rpm) during the invasion period increased invasion rates and subsequent parasitemia by 3.0 ± 0.4 fold (mean ± SEM, six experiments). These kinetics demonstrate that human-adapted *P*. *knowlesi* merozoites are more durable and have a significantly longer half-life of invasion capacity when compared to *P*. *falciparum in vitro*^29^.

### Development of a *P*. *knowlesi* merozoite invasion inhibition assay

**Figure 3:**
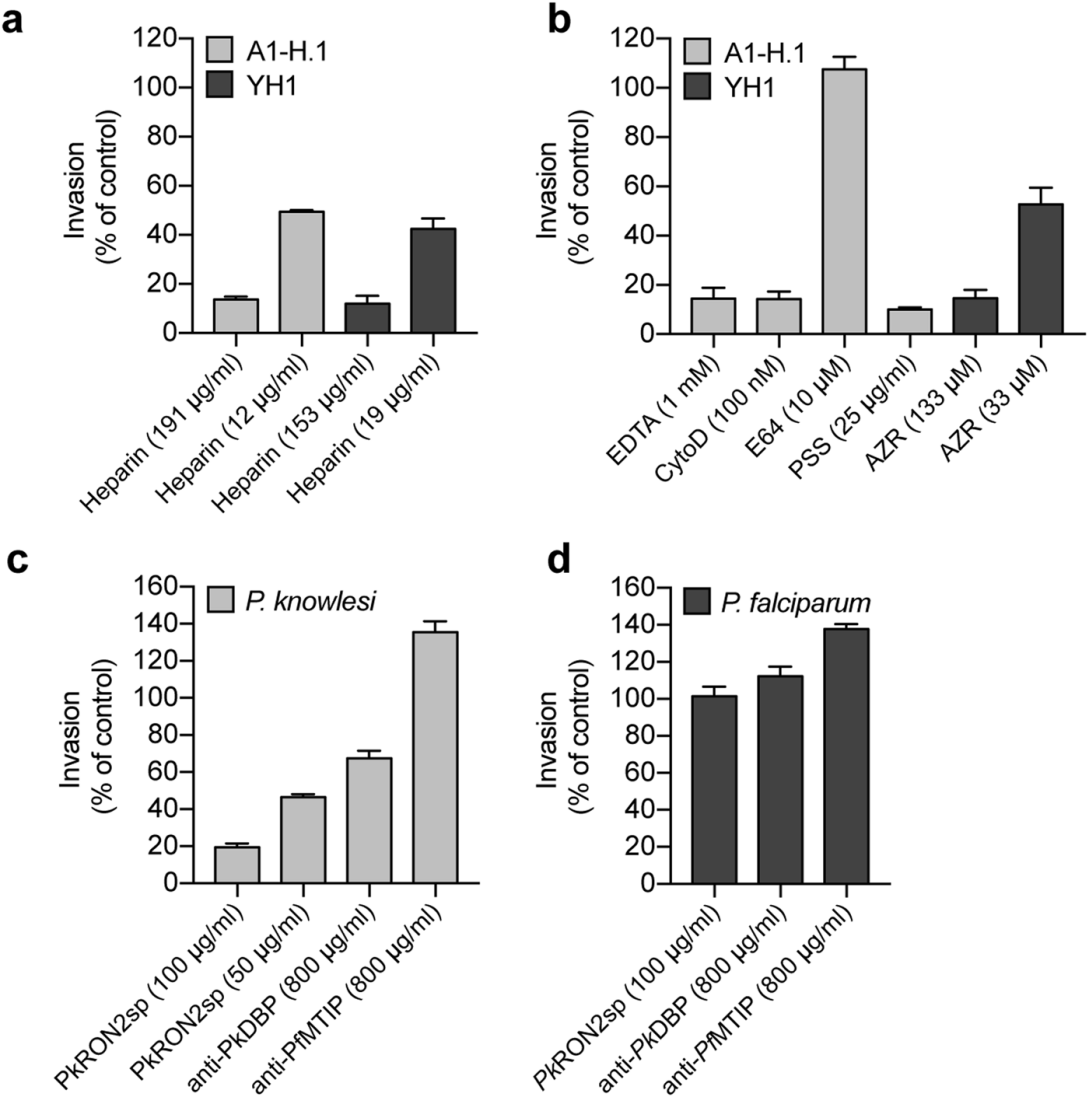
Development of a *P*. *knowlesi* invasion inhibition assay (*Pk*IIA). Panel of known inhibitory and non-inhibitory reagents was tested by *Pk*IIA using A1-H.1 and YH1 merozoites. (**a**) Heparin invasion inhibition of A1-H.1 and YH1 lines. Data are mean ± SEM of n = 3 assays in duplicate. Invasion is represented as a proportion of an untreated control. (**b**) All reagents were tested against A1-H.1 except for Azithromycin (AZR), which was tested against YH1. Invasion is represented as a proportion of an untreated control. Data are mean ± SEM of n = 3 assays in duplicate. The AZR YH1 data are mean ± range of n = 2 assays in duplicate. (**c**) *Pk*IIA and *Pf*IIA (**d**) can be used in tandem to show a specific peptide mimic, *Pk*RON2sp, and vaccine induced antibodies against *Pk*DBP give contrasting invasion inhibitory effects against *P*. *knowlesi* and *P*. *falciparum*. Invasion is represented as a proportion of an untreated control. Data are mean ± SEM of n = 3 assays in duplicate.

Erythrocyte invasion by *P*. *knowlesi* and *P*. *falciparum* merozoites share some mechanistic similarities^34^, but the erythrocyte receptor-parasite ligand interactions essential for entry differ dramatically between the species^3^. Drug molecules, peptides or antibodies that inhibit *P*. *falciparum* invasion are therefore likely to differ in their capacity to inhibit *P*. *knowlesi* invasion. To directly compare invasion-inhibitory molecules between the different species, we developed an invasion inhibitory assay (*Pk*IIA) for high-throughput testing of putative *P*. *knowlesi*-inhibitory reagents. Validating the assay, we confirmed activity of cytochalasin D (cytoD, 100 nM), the calcium chelator Ethylenediaminetetraacetic acid (EDTA, 1 mM) and the glycosaminoglycan polymer heparin (191 µg/ml), each inhibitory to *P*. *falciparum* merozoite invasion^12,13^ (Fig. 3a,b). The antibiotic Azithromycin (AZR), shown to have invasion inhibitory activity against *P*. *falciparum* merozoites *in vitro* with an IC_50_ of 10 μM^17^, also demonstrated an ability to inhibit *P*. *knowlesi* invasion, though only at a 4-fold higher concentration (IC_50_ of 40 μM) (Figs 3b and S4).

We next used the *Pk*IIA to explore the merozoite-RBC tight junction, an essential mechanistic interaction that organises the invasion process^35^. The strong binding interaction between the secreted apical membrane antigen (AMA)1 and rhoptry neck protein (RON)2 is highly conserved across *Plasmodium* and related apicomplexan parasite species^35^, and likely underpins the structure of the junction^36,37^. As expected, we found that a peptide (*Pk*RON2sp) encoding the short di-sulfate-bonded loop of *Pk*RON2 (orthologous to the region defined for *Pf*RON2 that interacts with *Pf*AMA1^38,39^) effectively inhibited *P*. *knowlesi* invasion (Fig. 3c) whilst the same polypeptide did not inhibit *P*. *falciparum* invasion (Fig. 3d). This supports expectations for the species-specific nature of the AMA1-RON2 interaction^38,39^.

Finally, we asked whether *Pk*IIA could be used to probe inhibition of merozoite invasion by *P*. *knowlesi -* specific inhibitory antibodies. Polyclonal serum raised against the essential *P*. *knowlesi* invasion protein *Pk*DBP-alpha^40^ (800 μg/ml, total IgG) successfully inhibited parasite invasion compared to an identically prepared non-specific polyclonal serum (anti-*Pf*MTIP also at 800 μg/ml total IgG) (Fig. 3c). The same anti-*Pk*DBP-alpha antibodies did not inhibit *P*. *falciparum* (Fig. 3d). Combined, these data validate key similarities and differences in signalling, mechanistic and receptor-ligand parasite interactions necessary for *Plasmodium* merozoite invasion of the RBC.

### Enrichment of *P*. *knowlesi* merozoite invasion events for microscopy analysis

**Figure 4:**
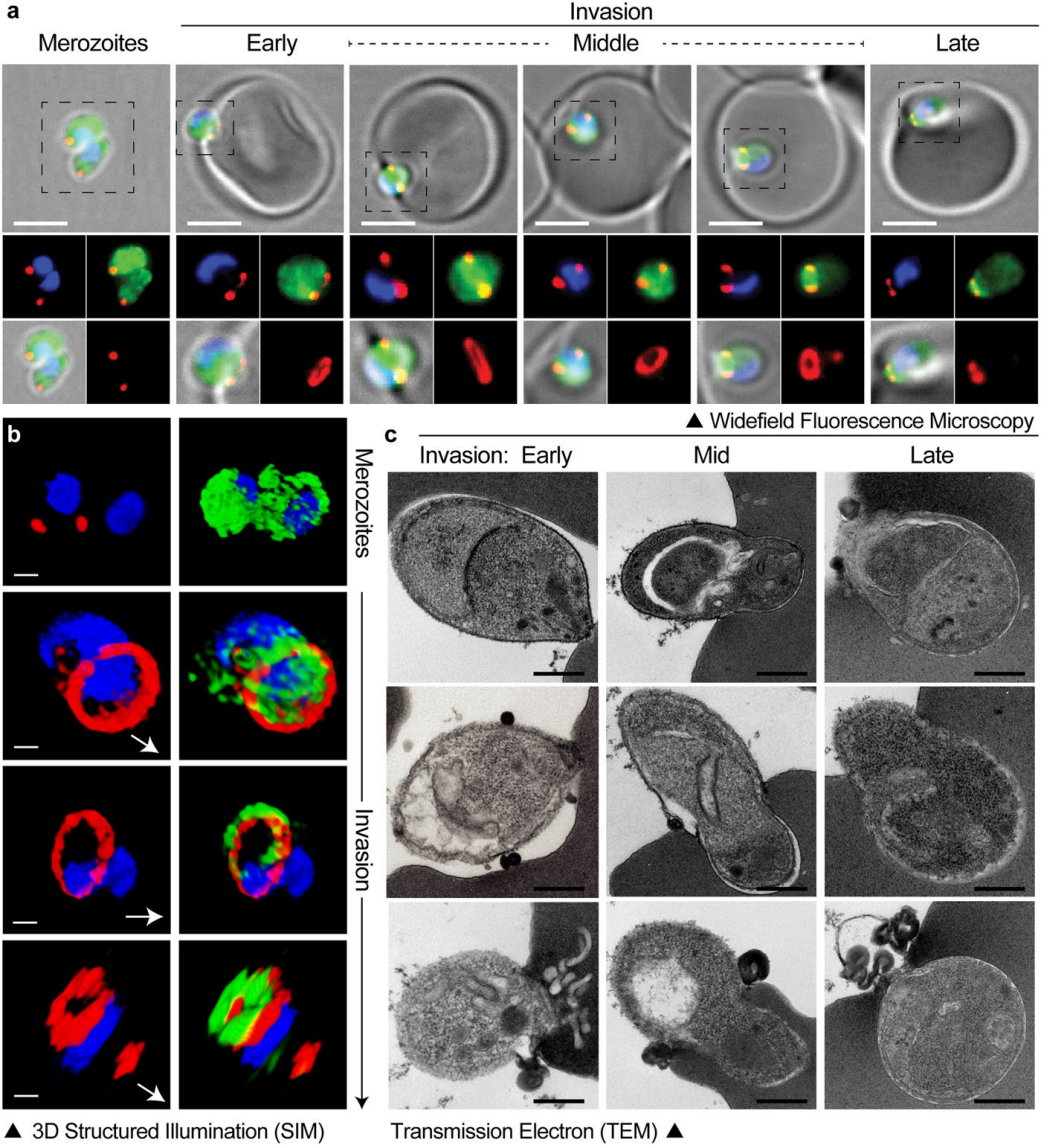
Imaging of merozoite invasion in fixed cells. Invading merozoites were visualized at all stages of invasion by IFA (**a**) 3D-SIM (**b**) and TEM (**c**). (**a**) Scale bars for IFA images are equal to 3 µm. Parasites are labeled with *Pk*RON2_mCherryHA, (red), parasite actin (green) and nucleus (blue). Insets (dotted box) shown below each respective image. Z-stack single slice red and blue merge (top left), z-stack single slice red and green merge (top-right), z-stack single slice red, green, blue and brightfield merge (bottom-left) and z-stack maximum intensity projection of red alone (bottom-right). (**b**) 3D-SIM of invading parasites. White arrows show direction of merozoite invasion. Scale bars = 0.5 µm. (**c**) Scale bars for TEM panels = 0.5 µm. For detailed parameters of imaging acquisition see supplemental methods.

Whilst older methods describe isolation of *P*. *knowlesi* merozoites^26–31^, the extended duration of the polycarbonate sieve process (over 2–3 hrs) or mechanical release of schizonts by syringe, both compromise parasite viability. Using such methods, and following fixation and sample preparation for electron microscopy (EM), capture of invading merozoites is consistently a rare event, making imaging and data collection a laborious process (L. Bannister, *personal communication*). Merozoites isolated by our filtration workflow could be readily captured at all stages of invasion and reliably imaged by immunofluorescence assay (IFA), 3D structured illumination super resolution microscopy (SIM) or EM (Fig. 4a–c). Importantly, invasion events could be enriched to a point where their frequency permitted facile image acquisition, making quantitative imaging of invasion possible at a level comparable to that with *P*. *falciparum*^41^.

Focussing attention on the parasite-RBC tight junction^35^, we developed a transgenic *Pk*RON2_mCherryHA A1-H.1 line by endogenous tagging of the RON2 genomic locus. *Pk*RON2_mCherryHA tagged merozoites could be fixed and visualized at different stages of the invasion process (early, middle and late invasion) by IFA and 3D-SIM (Fig. 4a,b). Pre-invasion, RON2 (red) was seen apically located on the merozoite, consistent with its rhoptry localisation in *P*. *falciparum*^23^. As junction formation proceeded, a ring of RON2 formed around the merozoite, migrating from apical to posterior (Fig. 4a,b) consistent with labelling in *P*. *falciparum*^23^. Parasite actin (green) of invading parasites was co-stained and seen to localise at or posterior to the tight junction as defined by the *Pk*RON2 ring (Fig. 4a,b), again consistent with that reported in *P*. *falciparum*^24^.

**Figure 5:**
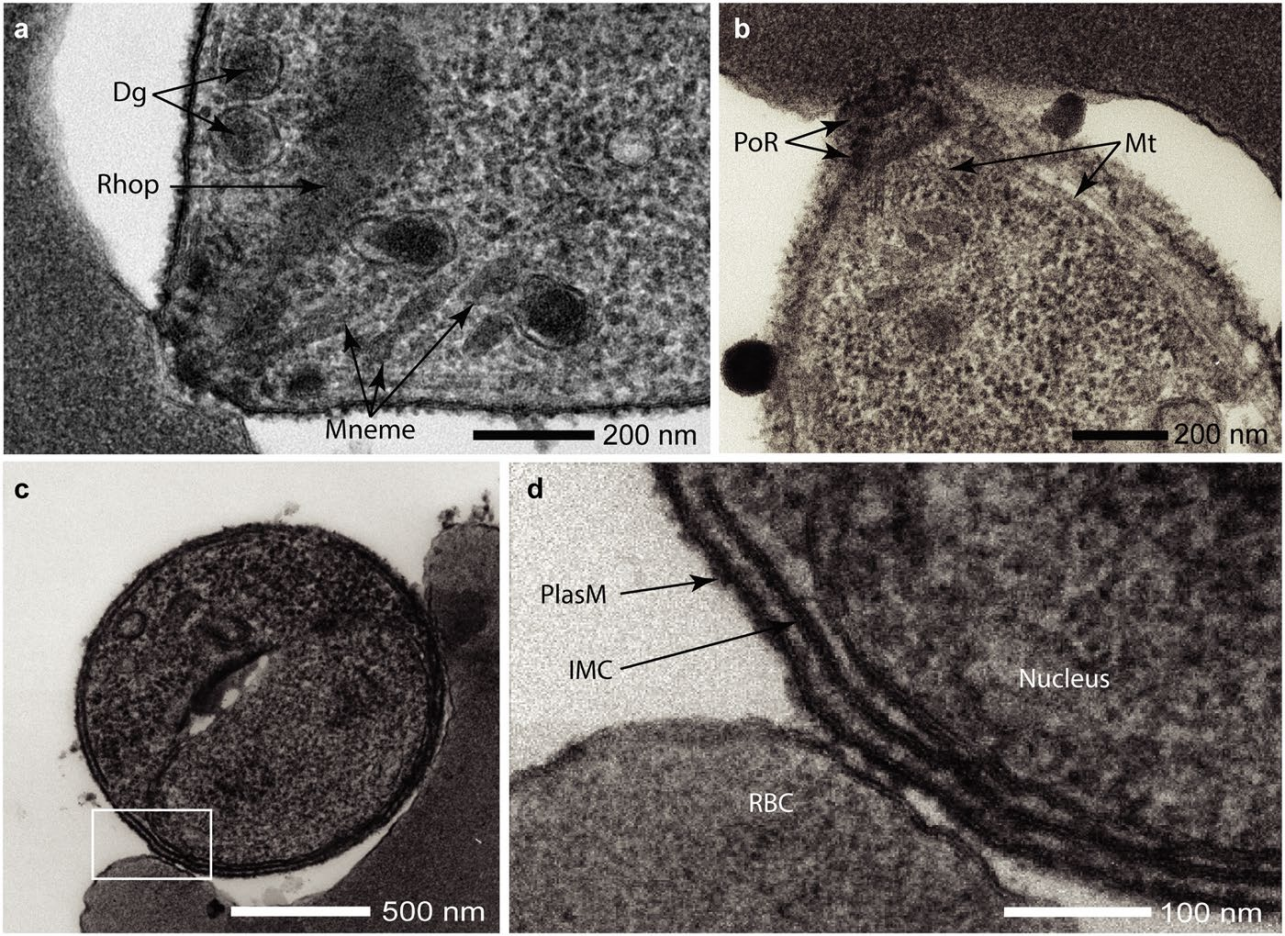
Transmission electron micrographs of invading *P*. *knowlesi* merozoites illustrating undamaged structures. (**a**) Apical end of a merozoite newly attached to a RBC prior to tight junction formation; dense granules (Dg), a rhoptry (Rhop) and micronemes (Mnme) are visible. (**b**) Apically attached merozoites sectioned to one side of the central axis and showing microtubules (Mt) and the polar rings (PoR) in the apical prominence. (**c**) Merozoite in the early stage of attachment prior to apical contact; the area within the box is enlarged in (**d**) to show normal structure, with the plasma membrane (PlasM) with its external coat and the inner membrane complex (IMC) overlying the nucleus, showing no loss of structural integrity. For detailed parameters of imaging acquisition see supplemental methods.

For high definition interrogation of *P*. *knowlesi* invasion, invading merozoites were also reliably captured and imaged by transmission EM (Fig. 4c). At 20,000x, organelles and cellular components of the merozoite were easily distinguished (Figs 4c and 5a–d). At higher magnifications, continuous, undisrupted membrane bilayers of the parasite plasma membrane, inner membrane complex and nuclear membrane were clearly observed (Fig. 5a–d), showing merozoite integrity being consistently maintained following double filtration.

### Polysulfonate tools for growth inhibition and synchronization of *P*. *knowlesi* merozoites

**Figure 6:**
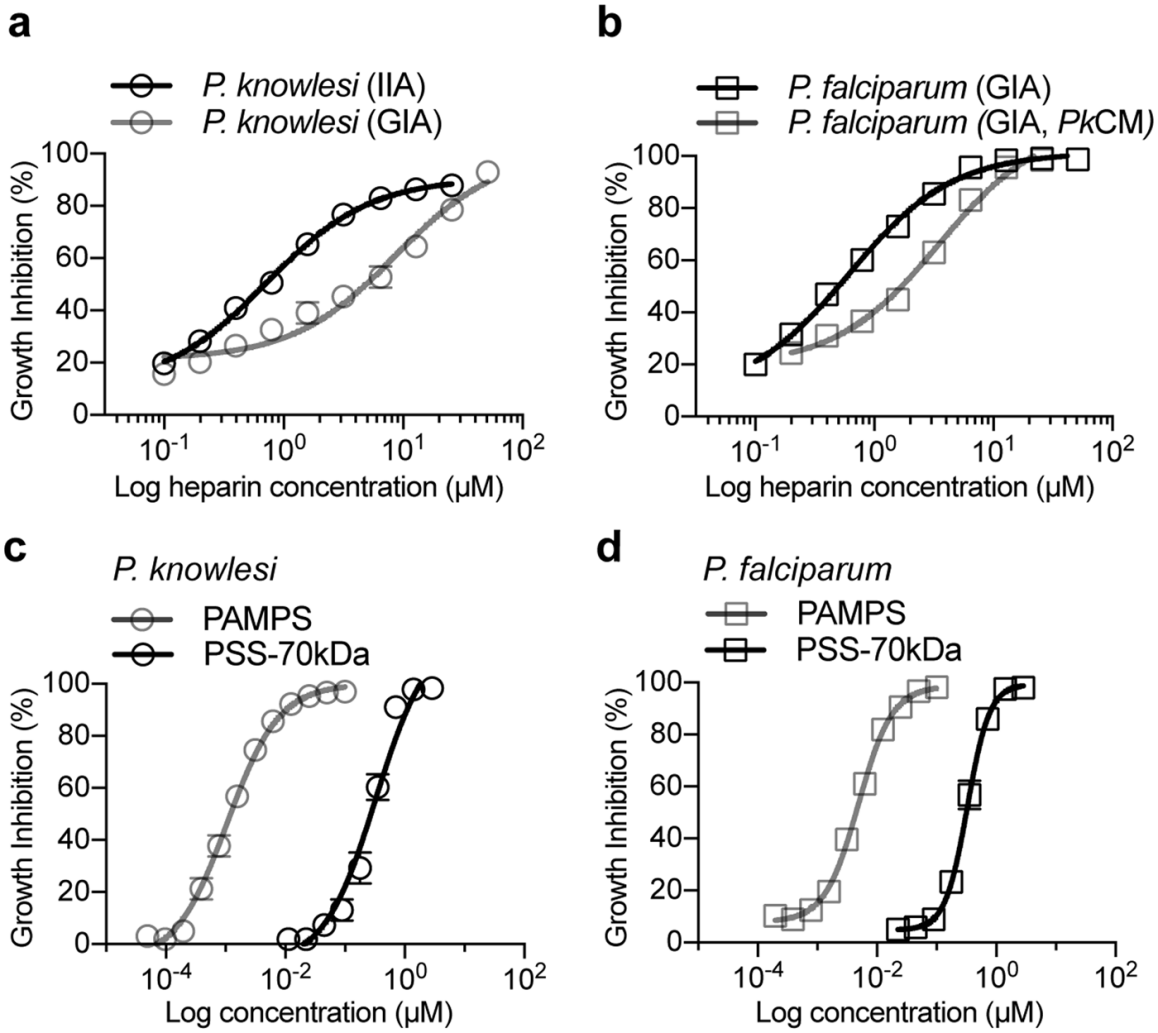
Polysulfonate molecules are effective growth inhibitors of *P*. *knowlesi* and *P*. *falciparum* under *in vitro* culture conditions. IC_50_ of heparin for both *P*. *knowlesi* (**a**) and *P*. *falciparum* (**b**) increases when equine serum is present in RPMI media. Data are mean ± SEM of n = 3 (*Pf*GIA and *Pk*GIA) or n = 2 (*Pf*GIA-*Pk*CM and *Pk*IIA) assays in duplicate. GIA = Growth Inhibitory Assay, IIA = Invasion Inhibitory Assay, *Pk*CM = *P*. *knowlesi* Complete Media. (**c**) *P*. *knowlesi* A1-H.1 and *P*. *falciparum* 3D7 (**d**) growth inhibition by synthetic polysulfonates. Data are mean ± SEM of n = 3 assays in duplicate (*P*. *knowlesi*) and mean ± range of n = 2 assays in triplicate (*P*. *falciparum*).

Having developed a robust platform for interrogating invasion, we finally sought to define novel inhibitors of invasion for use both in synchronising cultures but also as foundations for therapeutic development. Heparin has emerged as an important tool for synchronisation of blood stage *P*. *falciparum* cultures (in combination with sorbitol) because of its ability to inhibit merozoite attachment *in vitro*, enabling synchrony to a tight window of 2–4 hrs of blood-stage development, as well as providing a starting point to develop invasion inhibitory glycans^42,43^. However, in our attempts to mimic *P*. *falciparum* synchronization with *P*. *knowlesi* A1-H.1 and YH1 strains, heparin was unable to achieve tight synchrony of the A1-H.1 strain. Of note, the A1-H.1 line of *P*. *knowlesi* only exhibits normal asexual development in media with elevated serum content^7^. Elevated human serum requirements can be substituted with equine serum to maintain A1-H.1 *in vitro* cultures^11^. In contrast, the YH1 line, like *P*. *falciparum*, can be continuously cultured in media supplemented solely with 0.5% v/v Albumax II^9^. To explore whether the higher serum content might affect heparin efficacy versus biological differences between parasite strains, growth inhibition assays (GIA) with either strain were completed and compared to *P*. *falciparum* (Fig. 6a,b). The GIA IC_50_ of heparin against *P*. *knowlesi* YH1 parasites was 0.9 μM (13.69 μg/ml). This compared with a GIA IC_50_ of 8.69 μM (130 μg/ml) for A1-H.1 (Fig. 6a). However, when an IC_50_ was calculated using the *Pk*IIA, measuring merozoite invasion directly (as opposed to schizont through ring growth), the IC_50_ for A1-H.1 diminished to 0.72 μM (10.8 μg/ml) (Fig. 6a), in line with values obtained against *P*. *falciparum* 3D7 and *P*. *knowlesi* YH1. Minimal equine serum is present in the *Pk*IIA as merozoites are isolated in RPMI media only, suggesting that the serum interfered with heparin’s invasion inhibitory activity. This was confirmed by calculating a GIA IC_50_ for heparin against *P*. *falciparum* 3D7. When grown in A1-H.1 complete media containing 10% v/v equine serum, GIA IC_50_ increased nearly 6-fold from 0.63 μM (9.5 μg/ml) to 3.50 μM (52.5 μg/ml) (Fig. 6b). Similar reports of drug binding to serum proteins have also been observed in other *Plasmodium* growth assays^44^.

Since the YH1 line was sensitive to heparin under normal growth conditions, we assessed whether growth of this *P*. *knowlesi* line could be inhibited by a panel of heparin-like molecules recently identified as having activity against *P*. *falciparum* growth^42,43,45^. The growth inhibitory activity of the heparin-like molecules was similar at the concentrations tested between *P*. *knowlesi* YH1 and *P*. *falciparum in vitro* (Table S1), highlighting the potential to develop heparin-like molecules as anti-malarials with activity against multiple *Plasmodium* spp. Given limitations in being able to culture the A1-H.1 strain in low serum conditions, we sought to identify alternative invasion inhibitory molecules that could replace heparin as a common *P*. *knowlesi* synchronisation tool. We screened a small panel of commercially available soluble polymers for their ability to inhibit A1-H.1 merozoite invasion under *in vitro* growth conditions with equine serum present (Fig. S5). Two lengths of poly(sodium 4-styrenesulfonate) (PSS) were tested (PSS-70kDa and PSS-1MDa), poly(2-acrylamido-2-methyl-1-propanesulfonic acid) (PAMPS, 2 MDa), poly(acrylic acid) (PAA1, DDMAT terminated, 10 kDa) and poly(acrylic acid) (PAA2, 250 kDa) (Fig. S5). Both forms of PSS and PAMPS gave near complete inhibition of invasion at 200 μg/ml (Fig. S5). Comparatively, heparin only gave ~50% inhibition at 200 μg/ml (Fig. S5). Sulfate groups are essential for the invasion inhibitory activity of heparin-like molecules^42,43^ and the sulfonate groups of PSS and PAMPS mimic this activity. The carboxylic acid-based polymers of PAA1 and PAA2 did not exceed the inhibitory effect of heparin (Fig. S5). Importantly, neither PSS-70kDa nor PAMPS affected growth and development from rings through schizonts over 27 hrs and both polymers could be readily washed out after transient incubation to allow normal invasion rates in the following cycle. PSS-70kDa was shown to specifically inhibit invasion by *Pk*IIA (Fig. 3b). IC_50_ GIA curves, analogous to those using heparin, were completed for PSS-70 kDa and PAMPS against *P*. *knowlesi* A1-H.1, producing values of 330 nM and 1.1 nM respectively (Fig. 6c). Both compounds also inhibited *P*. *falciparum* by GIA, yielding IC_50_ values of 346 nM (PSS-70kDa) and 5.1 nM (PAMPS) (Fig. 6d). These values are significant improvement on the A1-H.1 GIA IC_50_ of heparin (~8.7 µM) and confirm increased invasion inhibitory activity of larger molecules^46^. PSS and PAMPS polymers represent a class of reagents that not only facilitate strain-independent *P*. *knowlesi* culture synchronization *in vitro* but, given their chemical tractability, have potential applications to develop scaffolds for pan-specific polymer-based invasion-inhibitory therapeutics^46^.

## Discussion

The key role *Plasmodium* blood stages play in malaria disease places the merozoite as a central focus for understanding how infection is established as well as being a prime target for interventions that stop parasite replication. Given the transient nature of the extracellular merozoite, however, it has proven challenging to study merozoite biology in most *Plasmodium* species. We have successfully developed here a methodological workflow for isolating viable, synchronous and invasive merozoites from *P*. *knowlesi,* a zoonotic parasite that causes severe pathogenic malaria. As well as interrogating the kinetics of *P*. *knowlesi* merozoite entry (for both human tissue-culture adapted *P*. *knowlesi* lines, A1-H.1 and YH1) and how this compares to those of *P*. *falciparum* (the leading cause of malaria-associated mortality), we have used this platform to identify commercially available polysulfonate polymers, PSS and PAMPS, as strong inhibitors of invasion, giving improved potential for parasite synchronisation and pan-specific therapeutic development.

An important observed difference in the invasion biology between *in vitro* isolated *P*. *knowlesi* and *P*. *falciparum* merozoites is a marked difference in the time period over which invasion events are finalised. *P*. *falciparum* completes 80% of the total invasion events within 10 mins of co-incubation with RBCs^12^, whereas we show it takes *P*. *knowlesi* 30 minutes before 90% of invasion events are finalised. This might be a human RBC specific effect, with *P*. *knowlesi* merozoites struggling to invade after co-incubation and requiring a longer period of time sampling the RBCs prior to commitment to complete invasion. If validated, this might explain why *P*. *knowlesi* in human RBCs has a lower *in vitro* multiplication rate (3–4 fold) compared to growth in *M*. *fascicularis* cells (7 fold), a preference retained even in the human adapted parasite lines^11^. Complementary experiments with A1-H.1 merozoites and *M*. *fascicularis* RBCs will confirm this hypothesis. Similar to published *P*. *falciparum* invasion kinetics^12^, we saw a linear relationship between invasion rate of purified *P*. *knowlesi* merozoites over incubation time. It is not clear whether this continuous invasive potential over time for a population of *Plasmodium* spp. merozoites is due to merozoite specific (such as the choice and readiness of invasion ligands) or host cell specific (such as limited availability of suitable RBCs for invasion) factors and this insight will be the topic of future studies.

Despite this extended invasive window, *P*. *knowlesi* merozoites are nonetheless able to rapidly enter RBCs immediately after isolation; after 90 seconds, merozoites are easily trapped at all stages of invasion. Imaging parasites caught in these stages confirmed *Pk*RON2 distribution at the ring-like tight junction previously observed in *P*. *falciparum*, *P*. *berghei* and *T*. *gondii*^36–39^ and the co-localised ring of actin at or anterior to the junction^24^. This presents opportunities to understand the host-parasite molecular interactions in detail and *in situ*, and, given their larger size, suggests further high resolution imaging of *P*. *knowlesi* merozoites will provide an important new platform for comparative apicomplexan junction biology, something that is challenging with *P*. *falciparum* merozoites^41^.

In addition to their applicability for imaging, access to purified merozoites facilitates development of a *Pk*IIA able to discriminate between inhibitory and non-inhibitory chemical ligands, antibodies and peptide mimics. Our identification of polysulfonate polymers suggests future screens will be readily possible, finding therapeutic candidates targeting all stages of the invasion process and, together with a similar assay for *P*. *falciparum*, allow comparative investigation of conserved and divergent aspects underpinning invasion of either species into the same RBCs. This is exemplified by our demonstration, combining imaging with *Pk*IIA, of the importance of the *Pk*RON2-*Pk*AMA1 interaction that clearly points to conservation of the interaction at the tight junction between these two molecules^39^. The absence of inhibition using *P*. *falciparum* merozoites and the *Pk*RON2 peptide suggests the molecular interaction between RON2 and AMA1 is species specific, as has been suggested^38,39^. A recent report using transgenic parasites, however, challenges this insight demonstrating functional conservation between *P*. *falciparum* AMA1 with that of *P*. *vivax*^47^. Follow up studies with the same domains of *Pv*RON2 should resolve whether this is a unique feature of these two parasites or whether other receptor-ligand interactions mitigate the interaction in the absence of peptide inhibition. Inclusion of macaque red cells will be an important future inclusion. Finally, advances in *Plasmodium* genetic modification strategies, not least CRISPR/Cas9^48^, with *Pk*IIA will undoubtedly provide a much-needed platform to evaluate key *P*. *vivax* invasion antigens on a *P*. *knowlesi* background, and to undertake high-throughput screening of invasion inhibiting entities against *P*. *vivax*.

In conclusion, this study not only presents the first comparative invasion study of *P*. *falciparum* and *P*. *knowlesi* merozoites into human RBCs, but also provides an important methodological platform to allow direct comparative analysis of RBC invasion for the only two tissue-culture adapted *Plasmodium* human infectious species. Given the close evolutionary relationship between *P*. *knowlesi* and *P*. *vivax* (geographically the most widespread human malaria parasite), and the absence of *in vitro* culture systems to explore therapeutics against it, *P*. *knowlesi* purified merozoites and the *Pk*IIA provide a much-needed avenue for investigating some of the key gaps in our understanding of human malaria parasite biology.

## Methods

### Parasite culture and synchronization

*P*. *knowlesi* A1-H.1 parasites (ICL) were cultured as described^11^. Parasites were synchronized to a 3.5 hr window by two Nycodenz gradients carried out 3.5 hrs apart. PSS to a final concentration of 200 µg/ml could replace the second Nycodenz gradient. *P*. *knowlesi* YH1 parasites (Adelaide) were cultured as described^9,49^. *P*. *knowlesi* YH1 parasites could be routinely synchronised to early ring stages with heparin^42^.

### *P*. *knowlesi* merozoite invasion assay

50–150 mL of tightly synchronized parasites (0–3.5 hr post-invasion), were allowed to develop from ring stages until majority were observed to be “teardrop” schizonts (Fig. S1) (23.5–26 hrs for A1-H.1; 26–29 hrs for YH1). Late-stage parasites were pelleted, washed once in incomplete media to remove debris and separated from uninfected RBCs by MACS magnet separation (CS column, Miltenyi Biotec). Parasites were returned to complete media at 37 °C and incubated with 10 μM E64 (Sigma-Aldrich) for <4 hrs. Pelleted E64-treated schizonts were resuspended in incomplete media (23 °C) to a 1.5–2% schizont v/v mixture and double filtered through a 3 μm filter (nucleopore etched membrane, Whatman 420200 and 110612) and then immediately a 2 μm filter (GMF media, Whatman 6783–2530). Removal of haemozoin was achieved by MACS magnet separation (LS column, Miltenyi Biotec). Filtrate, containing merozoites, was added to fresh RBCs pre-aliquoted in a 96-well plate in presence or absence of inhibitors as required. Plates were shaken at 750 rpm at 37 °C for 45 mins, supplemented with complete media containing heparin or PSS to prevent further invasion. The concentration of merozoites, RBCs and other cell populations was determined using Count-Bright Absolute Counting Beads according to the manufacturers’ protocol (Invitrogen)^50^.

### Immunofluorescence microscopy and electron microscopy

Immunofluorescence assay (IFA) and 3D-SIM imaging was achieved with PkRON2_mCherryHA A1-H.1 by adding merozoite filtrate to fresh RBCs (final hematocrit 0.5%), shaken at 1000 rpm, 37 °C for 90 seconds, fixed by addition of an equal volume of 2x fixative (8% paraformaldehyde/0.015% glutaraldehyde) (Sigma-Aldrich) and permeabilized with 0.1% Triton X-100/PBS for 10 mins. Samples were blocked with 3% BSA/PBS and incubated with anti-actin (5H3^24^) and anti-HA (3F10, Sigma-Aldrich) followed by Alexa 488 (anti-mouse) and Alexa 594 (anti-rat) conjugated secondary antibodies (Invitrogen, Thermo Fisher Scientific) respectively. Cells were smeared on slides and mounted in VectaShield (Vector Laboratories) with 0.1 ng/ml DAPI to label the parasite nucleus.

For EM visualization 1 ml of merozoite filtrate was added to 10–20 μl packed RBCs and shaken at 1000 rpm, 37 °C for 90 seconds. Cells were fixed in 2.5% glutaraldehyde, 0.75% tannic acid in 0.1 M sodium cacodylate at pH 7.1 for 3 hrs at room temperature and then washed three times, each for 20 mins in 0.1 M sodium cacodylate buffer. Cell pellets were left overnight at 4 °C. Samples were osmicated with 1% osmium tetroxide in 0.1 M sodium cacodylate for 1.5 hrs at room temperature (RT) and washed with 0.1 M sodium cacodylate. Samples were stained for 1 hr at RT with 1% w/v aqueous uranyl acetate, dehydrated in ethanol series and embedded in epoxy resin (TAAB). 90 nm sections were cut using Leica EM UC7 ultramicrotome and contrasted for 2 mins with Uranyless (TAAB) and 1 min with 3% Reynolds lead citrate (TAAB) according to manufacturer’s instructions. Samples were imaged on a JEOL JEM-1400Plus TEM (120 kV) fitted with a Ruby Camera (2 K × 2 K).

## Electronic supplementary material

Supplementary Information
