# Supplementary material for: Cellular dissection of malaria parasite invasion of human erythrocytes using viable Plasmodium knowlesi merozoites
Source: Sci Rep. 2018 Jul 5;8:10165. doi: 10.1038/s41598-018-28457-z (PMC6033891; doi:10.1038/s41598-018-28457-z)

SUPPLEMENTARY DATA FOR

Cellular dissection of malaria parasite invasion of human erythrocytes using viable *Plasmodium knowlesi* merozoites

Oliver Lyth[1], Gema Vizcay-Barrena[2], Katherine E. Wright[1], Silvia Haase[1], Franziska Mohring[3], Adrian Najer[1], Isabelle G. Henshall[4], George W. Ashdown[1], Lawrence H. Bannister[2], Damien R. Drew[5,6], James G. Beeson[5,6], Roland A. Fleck[2], Robert W. Moon[3], Danny W. Wilson[4,5]* and Jake Baum[1]*

[1] Department of Life Sciences, Imperial College London, Sir Alexander Fleming Building, Exhibition Road, South Kensington, London, UK. [2] Centre for Ultrastructural Imaging, Guy's Campus, Kings College London, London, UK. [3] Department of Immunology and Infection, London School of Hygiene and Tropical Medicine, London, UK. [4] Research Centre for Infectious Diseases, School of Biological Sciences, The University of Adelaide, Australia. [5] Burnet Institute, 85 Commercial Road, Melbourne, Victoria, Australia; and [6] Central Clinical School, Monash University, Victoria, Australia

*Correspondence to:

Jake Baum | jake.baum@imperial.ac.uk

Danny W. Wilson | danny.wilson@adelaide.edu.au

This PDF file includes:

Supplemental Methods

Table S1

Figs. S1 to S5

SUPPLEMENTAL METHODS

Reagents

Reagents tested by *Pk*IIA were acquired as follows; Heparin (Roche and Fannin), CytoD, EDTA, E64 and Azithromycin (all Sigma). *Pk*RON2sp was a kind gift from D. Drew and J. Beeson. To investigate the inhibitory capacity of the *Pk*RON2 peptide homologue, we had the *Pk*RON2 peptide (Asp-1975 to Thr-2013) synthesized with cyclized cysteine residues to form a disulfide bonded β-hairpin loop. To generate antibodies against *Pk*DBP and *Pf*MTIP, recombinant protein was expressed and purified as described [1,2]. Rabbits were immunized and serum collected after 28 days (Eurogentec). Serum was dialyzed against PBS pH7.4 and total rabbit IgG purified using a HiTrap Protein A HP 5ml column (GE Healthcare). Sodium forms of polymers, PSS-70kDa (Sigma, 243051) and PSS-1MDa (Sigma, 527491) were directly dissolved in PBS and sterile filtered. Acidic forms of polymers PAMPS (Sigma 191973), PAA1 (Sigma, 775843) and PAA2 (Sigma, 416002) were first dissolved in phosphate buffer and buffer exchanged on a PDMiniTrap column (Sephadex G25, GE) using PBS and sterile filtered.

Parasite culture and synchronization

P. knowlesi A1-H.1 parasites (Imperial College London) were cultured as described [3] in human O^+ RBCs in RPMI-HEPES medium supplemented with 2.3 g/L sodium bicarbonate, 2 g/L dextrose, 0.05 g/L hypoxanthine, 0.025 g/L gentamicin, 0.292 g/L L-glutamine, 5 g/L Albumax II (Gibco) and 10% (v/v) equine serum [3] (Life Technologies). Parasites were cultured at 37°C with a gas mixture of 90% N_2, 5% O_2, 5% CO_2. Parasites were synchronized to a 3.5 hr window by two Nycodenz gradients carried out 3.5 hrs apart. The second Nycodenz gradient can be replaced by addition of PSS to a final concentration of 200 µg/ml.

P. knowlesi YH1 parasites (the University of Adelaide) were cultured as described [4,5] in human O^+ RBCs in RPMI-HEPES culture medium (pH 7.4) supplemented with 0.007 g/L

hypoxanthine, 2.1 g/L $NaHCO_3$, 0.01 g/L gentamicin and 0.5% Albumax II (Gibco, Melbourne, VIC, Australia). YH1 parasites were cultured at 37°C in an atmosphere of 1% O_2, 4% CO_2 and 95% N_2. *P. knowlesi* YH1 parasites could be routinely synchronised to early ring stages using heparin synchronization [6].

P. knowlesi merozoite invasion assay and growth inhibition assays

To observe the "teardrop" morphology of schizonts, methanol fixed, air-dried smears were Giemsa stained briefly for up to 10 seconds.

To count the different cell populations, an identical volume of CountBright Beads was added to 3x concentration SybrGreen (Thermo Scientific) stained pre-filtration, 3 µm filtrate and double filtrate samples. Samples were stained for 20 mins. Samples were then mixed thoroughly and counted using a BectonDickinson LSR Fortessa II flow-cytometer with a minimum of 2,000 bead counts collected per sample. The different cell populations were analyzed using FlowJo software (Tree Star). Forward scatter (FSC-H) gives an indication of the size of a cell and FITC-H (SybrGreen staining) gives an indication of the amount of DNA. Late-stage parasites are large and contain multiple genome copies (FSC high/FITC high), segmented schizonts contain multiple genome copies but are slightly smaller than non-segmented late-stage parasites as the RBC membrane collapses around the segmented schizont (FSC mid-high/FITC high) and merozoites are small and only contain one copy of the genome (FSC low/FITC low). Countbright beads were gated using a FITC high/FL-2 high gate. Invasion rates were calculated as percentage of RBCs invaded x [(RBCs per µl)/(merozoites per µl)] [7].

Filtrate, containing merozoites, was added immediately to fresh RBCs pre-aliquoted in a polystyrene 96-well plate in presence or absence of invasion inhibitors as required. For *Pk*IIA, final volume per well was 40 µl (16 µl 1.25% hematocrit in incomplete media, 4 µl 10x concentration invasion inhibitor and 20 µl merozoite filtrate). Plates were shaken at 500 -

750 rpm at 37ºC for 30 mins, and then complete media supplemented with heparin or PSS was added to each well to prevent further invasion and parasites allowed to develop for 16-20 hrs in a gassed box at 37°C. For *Pk*IIA, after the 30-minute invasion period, RBCs were washed twice with incomplete media to remove inhibitors and then returned to culture.

To define the kinetics of invasion, E64-treated schizonts were resuspended and filtered as described above and 30 µl merozoite filtrate was added to 10 µl pre-aliquoted 2% hematocrit RBCs in 96 well plates. The plate was returned to 37°C and agitated. At regular time points 10 µl of PSS (1000 µg/ml) was added to each well containing 40 µl of RBCs + merozoite filtrate. After 45 mins, the final time point, cells were incubated at 37°C and resulting parasitemias were analyzed 16-20 hrs later by flow cytometry.

For growth inhibition assays, synchronous *P. knowlesi* parasites at 0.5-1.0% parasitemia and 2% hematocrit in parasite culture media were added to wells of a 96-well plate in a volume of 100 µl culture. A two-fold series dilution of each invasion inhibitor was performed across the plate. Parasitemia was assessed after 20 hrs.

Parasitemias were determined by staining with SybrGreen and analyzed by flow cytometry. To determine invasion rates and invasion inhibition by *Pk*IIA, parasitemia was calculated first by gating for singlet RBCs (FSC-A/FSC-W) and then infected FITC-positive cells (FSC high/FITC high). Parasitemia as calculated by *Pk*IIA was expressed as a percentage growth of non-inhibitory control wells and parasitemia as calculated by GIA was expressed as a percentage of untreated control wells.

Immunofluorescence microscopy and electron microscopy

Immunofluorescence assay (IFA) images were acquired with a Nikon plan apo γ 100x/1.45 oil immersion lens on a Nikon eclipse T*i* microscope and images processed with NIS Elements (Nikon). 3D-SIM images were acquired using the Zeiss Elyra PS1 system (Carl Zeiss) through a 63× Plan Apochromat 1.4NA oil objective (Carl Zeiss), a 1.6× magnification

lens was placed in front of the PCO.edge camera (PCO, Germany) for a pixel size of 64 nm. A CoolLED pE-300 (CoolLED) was used to locate fluorescent parasites by eye before switching to the SIM mode where 405, 488 and 561 nm lasers were used to capture data, using the appropriate filter cubes. The structured illumination pattern was generated by passing the 405, 488 and 561 nm lasers respectively through a 23, 28 and 34 μm diffraction grating. The illumination pattern was laterally shifted through 5 phases, before passing through z to capture a full stack; this was repeated through the 3 rotations to ensure homogeneous resolution enhancement. Images were visually inspected to ensure minimal bleaching occurred during acquisitions. Laser powers were set to 5 % for the 405 nm channel and 2 % for 488 and 561 nm channels, with acquisition frame rates of 100, 200 and 100 ms respectively. This raw data was then reconstructed, using the Auto noise filter and setting the output to Raw Scale, all other settings were kept as default. The reconstructed pixel sizes for the x, y plane were 32 nm.

***P. knowlesi* A1-H.1 PkRON2_mCherryHA line**

DNA constructs for modifying the *RON2* locus of A1-H.1 parasites were based on a modified *Pk*conGFPep vector (kind gift of Robert Moon [8]). *Pk*conGFPep was linearized with NotI and SacII. Gibson cloning [9] was performed with linearized *Pk*conGFPep backbone, *Pk*RON2 flank region PCR product amplified from A1-H.1 genomic DNA with primer pair RON2flank_fwd AATACTC**GCGGCCGC**CAGAGCAAGAAAGGTTAGAGC/RON2flank_rev CCTCCTCTCCCTTGCTCAC**ACCGGT**ACCCATCTGTATGCGGGCG), a synthetic gene sequence coding for the mCherryHA tag and PCR product encoding an in frame T2A skip peptide and neoR cassette amplified from vector pSLI (a kind gift of Tobias Speilmann, BNI) with primer pair T2AneoR_fwd ATGACGATAAG**GGTACC**GGAGAAGGAAGAGGAAGTTTATTAAC/T2AneoR_rev GCTGCCATAT**CCGCGG**TTAGAAGAACTCGTCAAGAAGG. Vector was linearized before parasite transfection with a unique Xma1 restriction site. A1-H.1 parasites were transfected

as described [8]. In brief, tightly synchronized mature schizonts were obtained and transfections were carried out using the Amaxa 4D electroporator (Lonza) and the P3 Primary cell 4D Nucleofector X Kit L (Lonza). Transfectants were selected by treatment with 100 nM pyrimethamine for one week and successful integration was subsequently shown by PCR genotyping with PCR pairs P1/P2 and P3/P4 (P1-CGAGGGAAATTATTCAAAACGC, P2-GGTCACCTTTAACTTGGCG, P3-CTTCCGGCTCGTATGTTG, P4-GGGCAACTGGGTAGTAAACC).

SUPPLEMENTAL REFERENCES

1 Singh, S. K., Hora, R., Belrhali, H., Chitnis, C. E. & Sharma, A. Structural basis for Duffy recognition by the malaria parasite Duffy-binding-like domain. *Nature* **439**, 741-744 (2006).

2 Douse, C. H. *et al.* Regulation of the Plasmodium motor complex: phosphorylation of myosin A tail-interacting protein (MTIP) loosens its grip on MyoA. *J Biol Chem* **287**, 36968-36977 (2012).

3 Moon, R. W. *et al.* Normocyte-binding protein required for human erythrocyte invasion by the zoonotic malaria parasite Plasmodium knowlesi. *Proc Natl Acad Sci U S A* **113**, 7231-7236 (2016).

4 Dankwa, S. *et al.* Ancient human sialic acid variant restricts an emerging zoonotic malaria parasite. *Nat Commun* **7**, 11187 (2016).

5 Lim, C. *et al.* Expansion of host cellular niche can drive adaptation of a zoonotic malaria parasite to humans. *Nat Commun* **4**, 1638 (2013).

6 Boyle, M. J., Richards, J. S., Gilson, P. R., Chai, W. & Beeson, J. G. Interactions with heparin-like molecules during erythrocyte invasion by Plasmodium falciparum merozoites. *Blood* **115**, 4559-4568 (2010).

7 Johnson, J. G., Epstein, N., Shiroishi, T. & Miller, L. H. Factors affecting the ability of isolated Plasmodium knowlesi merozoites to attach to and invade erythrocytes. *Parasitology* **80**, 539-550 (1980).

8 Moon, R. W. *et al.* Adaptation of the genetically tractable malaria pathogen Plasmodium knowlesi to continuous culture in human erythrocytes. *Proc Natl Acad Sci U S A* **110**, 531-536 (2013).

9 Gibson, D. G. *et al.* Enzymatic assembly of DNA molecules up to several hundred kilobases. *Nat Methods* **6**, 343-345 (2009).

10 Boyle, M. J. *et al.* Identification of Heparin Modifications and Polysaccharide Inhibitors of Plasmodium falciparum Merozoite Invasion That Have Potential for Novel Drug Development. *Antimicrob Agents Chemother* **61** (2017).

11 Chen, J. H., Lim, J. D., Sohn, E. H., Choi, Y. S. & Han, E. T. Growth-inhibitory effect of a fucoidan from brown seaweed Undaria pinnatifida on Plasmodium parasites. *Parasitol Res* **104**, 245-250 (2009).

SUPPLEMENTAL TABLES

Table S1: Growth inhibition of heparin-like compounds against *P. knowlesi* YH1 parasites compared to published *P. falciparum* growth inhibition[a] *in vitro*.

**Compound**<br><br>***100 μg/mL***	***P. knowlesi* YH1 growth inhibition *Mean[a] (SD)***	***P. falciparum* growth inhibition *Mean[a] (SEM)***
Bovine Lung Heparin	82 (0.1)	74 (5)[b]
MH of 5 kDa gc	81 (2.3)	73 (8)[b]
MH of 3 kDa	41 (13)	37 (0)[b]
MH of 3 kDa gc	55 (11)	70 (8)[b]
Enoxaparin	35 (4.9)	64 (5)[b]
Fucoidin	89 (9.5)	~95[c]
50 μg/mL		
K5-NSOS-H	94 (5)	~75[d]
K5-OS-H	76 (5.4)	<90[d]

[a] Growth inhibition (% of non-inhibitory control)
[b] From Boyle *et al*. Antimicrobial Agents and Chemotherapy, 2017 [10].
[c] From Chen *et al*. Parasitology Research, 2009 [11].
[d] From Boyle *et al*. Blood, 2010 [6].

Abbreviations: MH- porcine mucosal heparin; gc- glycol splitting.

SUPPLEMENTAL FIGURE LEGENDS

Figure S1. Framework to isolate viable *P. knowlesi* merozoites. Tightly synchronized late schizont stage *P. knowlesi* parasites after magnet purification (A), E64 incubation with schizonts (B), double filtration (C) and haemozoin removal (D) results in high purity. (E) Merozoite filtrate was passed over a small magnet column (Miltenyi) to rebind unruptured schizonts and free haemozoin crystals. Awareness of non-merozoite events allowed tighter gating of the merozoite population and more accurate calculation of merozoite:RBC ratios.

Figure S2. A1-H.1 merozoites purified by filtration exhibit normal development after invasion. Representative Giemsa stained infected RBCs during invasion (merozoite), one hr post invasion (ring) and 12 hrs post invasion (trophozoite) show highly synchronous cultures.

Figure S3. Uninfected RBC population is identified by flow cytometry to calculate RBC concentration and subsequent merozoite:RBC ratios.

Figure S4. *P. knowlesi* YH1 invasion inhibition by Azithromycin. Data are mean ± range of two assays.

Figure S5. Polysulfonate molecules tested for growth inhibition activity. (A) A panel of commercially available charged soluble polymers were tested for invasion inhibitory activity against A1-H.1 by GIA. Data are mean ± range of two assays in duplicate. Poly(sodium 4-styrenesulfonate) (PSS) was tested (PSS-70kDa and PSS-1MDa), along with poly(2-acrylamido-2-methyl-1-propanesulfonic acid) (PAMPS, 2MDa), poly(acrylic acid) DDMAT terminated (PAA1, 10kDa) and poly(acrylic acid) (PAA2, 250kDa). (B) Molecular structures of heparin, PSS, PAMPS, PAA1 and PAA2.

Lyth et al Figure S1

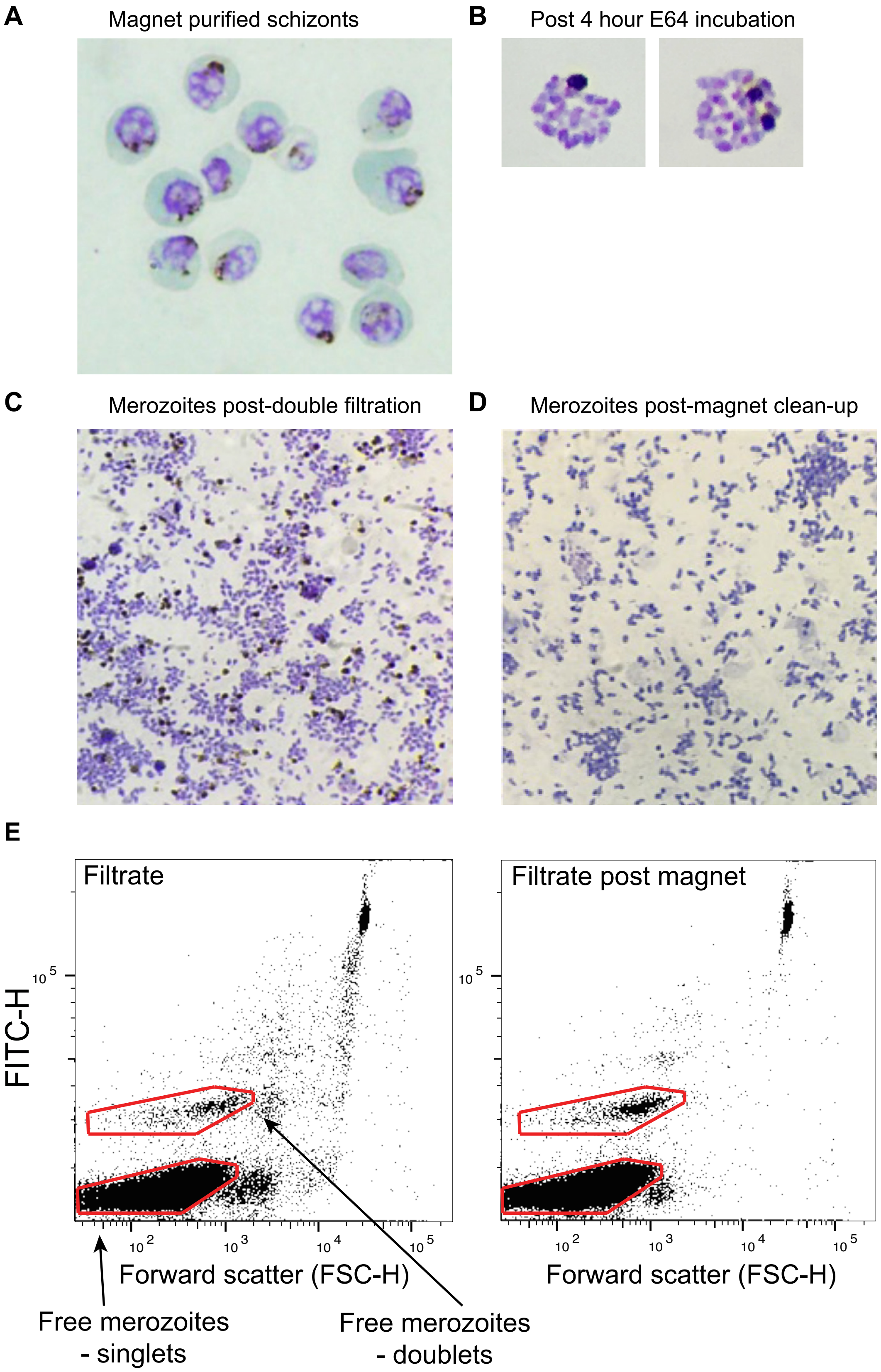

Lyth et al Figure S2

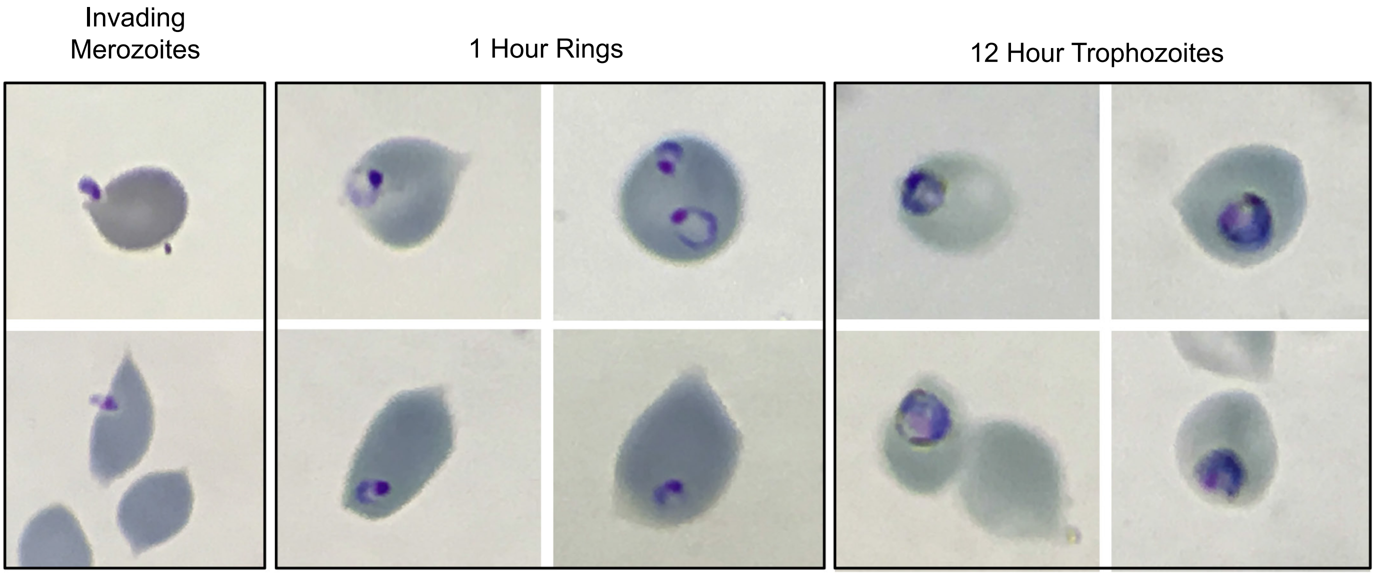

Lyth et al Figure S3

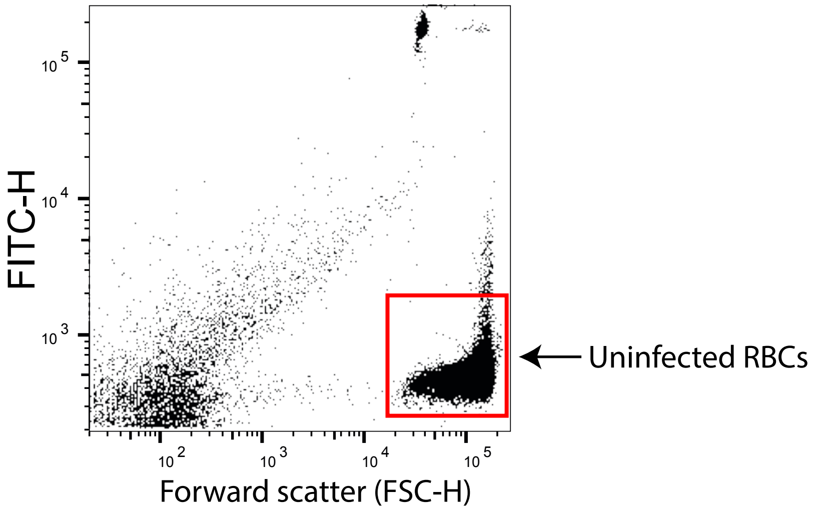

Lyth et al Figure S4

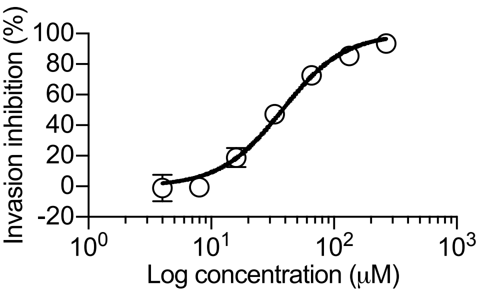

Lyth et al Figure S5

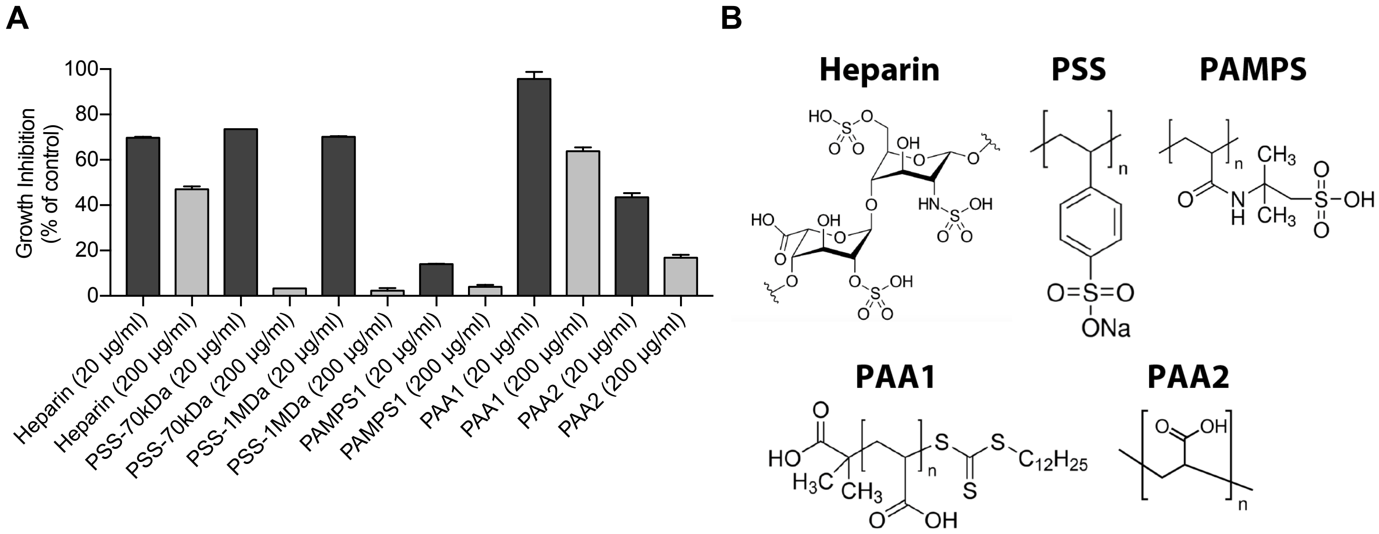

Supplement: Supplementary file 1 — Supplementary Information [file 41598_2018_28457_MOESM1_ESM.pdf]
